# Oxysterol research: a brief review

**DOI:** 10.1042/BST20180135

**Published:** 2019-04-01

**Authors:** William J. Griffiths, Yuqin Wang

**Affiliations:** Swansea University Medical School, ILS1 Building, Singleton Park, Swansea SA2 8PP, Wales, U.K.

**Keywords:** cholesterol, cytochrome p450, Hedgehog, Huntington's disease, metastasis, sterols

## Abstract

In the present study, we discuss the recent developments in oxysterol research. Exciting results have been reported relating to the involvement of oxysterols in the fields of neurodegenerative disease, especially in Huntington's disease, Parkinson's disease and Alzheimer's disease; in signalling and development, in particular, in relation to Hedgehog signalling; and in cancer, with a special focus on (25R)26-hydroxycholesterol. Methods for the measurement of oxysterols, essential for understanding their mechanism of action *in vivo*, and valuable for diagnosing rare diseases of cholesterol biosynthesis and metabolism are briefly considered.

## Introduction

**Figure 1.**
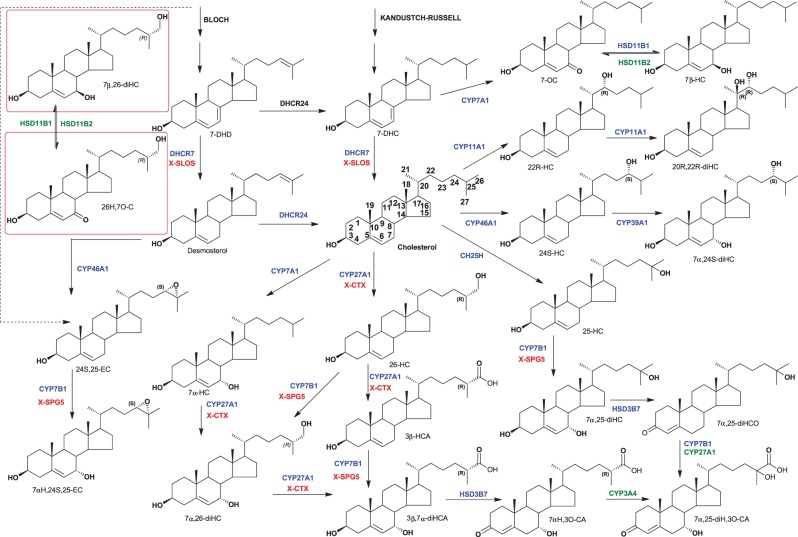
Structures of cholesterol, some of its precursors and some oxysterols. Once 7α-hydroxylated, oxysterols become substrates for the enzyme HSD3B7 (3β-hydroxysteroid dehydrogenase type 7) and can be converted from their 3β-hydroxy-5-ene to 3-oxo-4-ene forms. Shown in the red boxes are 7β,26-diHC and 26H,7O-C, assumed to be interconverted by the HSD11B enzymes. Enzymes, supported by experimental evidence are shown in blue, where activity is assumed enzymes are in green. Diseases resulting from enzymes deficiency are in red and indicated by an X preceding the disease abbreviation.

Oxysterols are oxidised forms of cholesterol or of its precursors (Figure 1) [1]. They include 1α,25-dihydroxyvitamin D_3_, the biologically active form of vitamin D_3_, 24S-hydroxycholesterol (24S-HC), also known as cerebrosterol, the major cholesterol metabolite found in the brain [2], 25-hydroxycholesterol (25-HC) synthesised in macrophages as a result of bacterial or viral infection [3,4] and 22R-hydroxycholesterol (22R-HC), the first metabolite of cholesterol in the steroid hormone biosynthesis pathway [5]. 7α-Hydroxycholesterol (7α-HC) is the first member of the neutral pathway of bile acid biosynthesis and (25R)26-hydroxycholesterol (26-HC also known as 27-hydroxycholesterol, when C-25 is asymmetric stereochemistry is assumed to be 25R unless stated otherwise, see Supplementary Table S1 for a list of common and systematic names) is the first member of the acidic pathway of bile acid biosynthesis [6]. In this review, we also include cholestenoic acids, where the terminal carbon of the cholesterol side-chain has been oxidised to a carboxylic acid, in the ‘family’ of oxysterols (Figure 1). In the present study, we will adopt the nomenclature system outlined by the Lipid Maps consortium [7] and follow IUPAC naming rules regarding oxidation of the terminal carbon of the sterol side-chain [8].

Oxysterols are bioactive molecules. The side-chain oxysterols, where oxidation has occurred in the sterol side-chain, are established ligands to the liver X receptors (LXRα, NR1H3; LXRβ, NR1H2) [9], inhibitors of the processing of SREBP-2 (sterol regulatory element-binding protein-2) to its active form as the master transcription factor for expression of genes in the mevalonate pathway of cholesterol biosynthesis [10] and potent allosteric modulators of the *N*-methyl-d-aspartate (NMDA) receptors [11,12]. The side-chain and ring doubly oxidised cholesterol metabolites 7α,25-dihydroxycholesterol (7α,25-diHC) and 7α,(25R)26-dihydroxycholesterol (7α,26-diHC, also called 7α,27-dihydroxycholesterol) are ligands to the G protein-coupled receptor (GPCR) 183, also known as the Epstein–Barr virus-induced gene 2 (EBI2), and guide immune cell migration of EBI2-expressing cells [13,14]. (25R)26-Hydroxy-7-oxocholesterol (26H,7O-C, also called 7-keto-27-hydroxycholesterol), 7β,(25R)26-dihydroxycholesterol (7β,26-diHC, also called 7β,27-dihydroxycholesterol), like the elusive 20S-hydroxycholesterol (20S-HC), and also cholesterol, are ligands to Smoothened (SMO), a key protein of the Hedgehog (Hh) signalling pathway, important for proper cell differentiation in embryonic tissue, and when misfunctioning can lead to basal cell carcinoma in adults [15–17].

Measurement of oxysterols, including C_27_ acids, in the circulation or cerebrospinal fluid (CSF) is of value for the diagnosis of rare inborn errors of cholesterol transport, biosynthesis and metabolism [18–25]; additionally, these measurements may have value for defining biomarkers of disease progression, particularly important in the development of new therapeutics for neurodegenerative disease.

## Measurement of oxysterols

**Figure 2.**
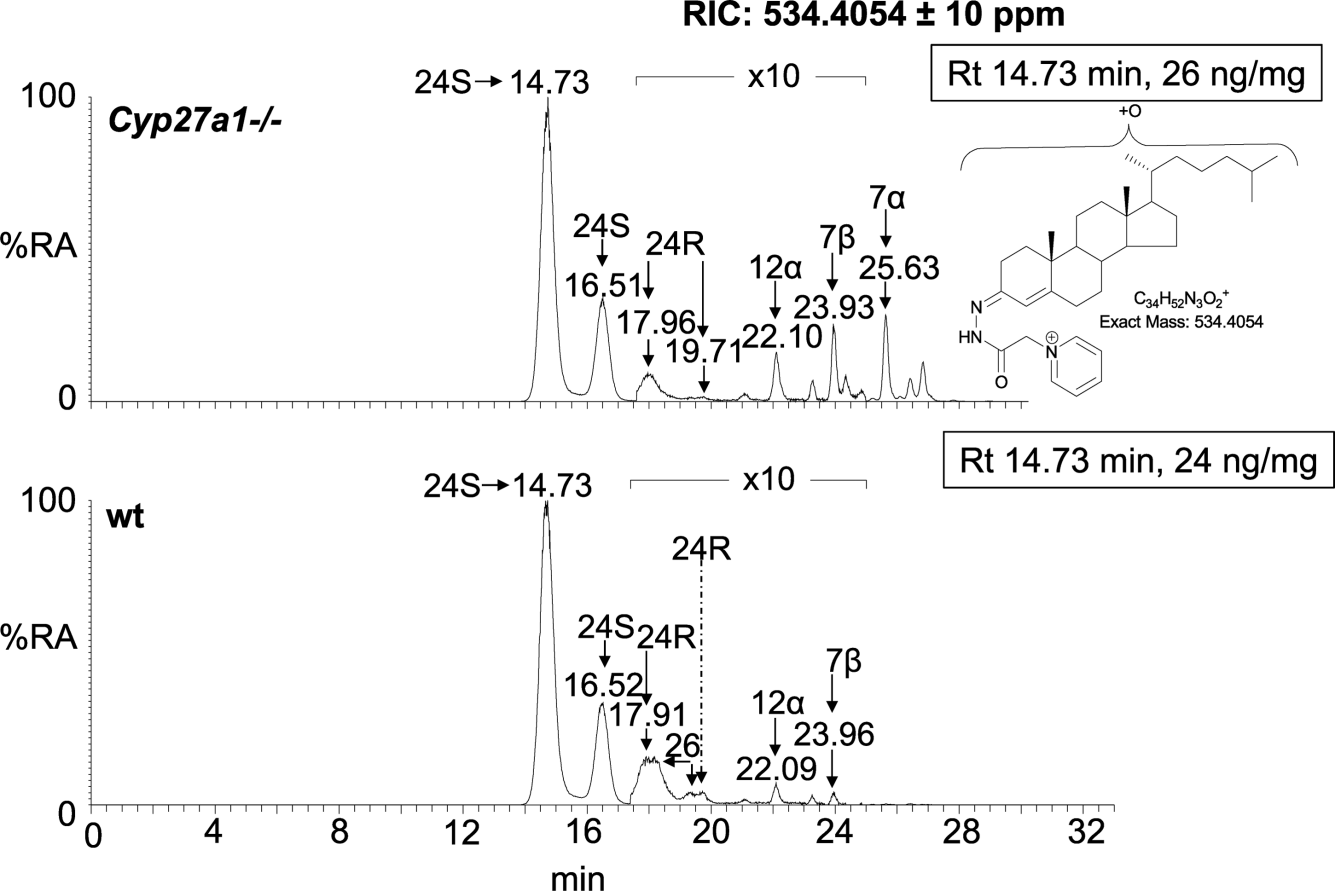
Chromatographic separation of mono-hydroxycholesterols, extracted from mouse brain, as Girard P derivatives demonstrating the complexity of the oxysterol profile. The different oxysterols are labelled with the location of the extra hydroxy group. No authentic standard was available for the oxysterol indicated to be 12α-hydroxycholesterol (12α-HC). As a consequence of derivatisation, each oxysterol may give twin peaks. The top panel shows mono-hydroxycholesterols in the brain from the *Cyp27a1* knock-out mouse, the bottom panel is from a wild-type mouse. The *Cyp27a1* knock-out mouse biochemically mimics the human disorder CTX, showing an absence of 26-HC and elevation in 7α-HC [30]. In both chromatograms, the *y*-axis is normalised to the most intense peak at 100% relative abundance (RA). The measured concentration of 24S-HC corresponding to this peak is given in the right-hand corner of each chromatogram. A hydrolysis step was not performed. To generate the reconstructed-ion chromatogram, mass spectrometry data were recorded at high resolution with an Orbitrap analyser. Reproduced from ref. [30].

Oxysterols are usually measured by gas chromatography–mass spectrometry (GC–MS) methods incorporating selected-ion monitoring [26,27] or liquid chromatography tandem–mass spectrometry (LC–MS/MS) methods exploiting multiple reaction monitoring [28]. When an alkaline hydrolysis step is included, the total oxysterol (non-esterified plus esterified) is measured. In the absence of hydrolysis, the non-esterified or ‘free’ molecules are measured. While derivatisation is a pre-requisite for GC–MS studies, this is not so for LC–MS/MS studies, although many workers have employed derivatisation to enhance sensitivity [19,21,22,29,30]. The extreme diversity of isomeric oxysterols makes chromatographic separation challenging; this problem is accentuated by the similar fragmentation patterns, in both GC–MS and LC–MS/MS of many epimers [30,31] (Figure 2). Besides chromatographic separation, avoidance of *ex vivo* oxidation of sterols, especially cholesterol, to produce non-endogenous oxysterols is another major challenge, particularly if cholesterol is not separated from endogenous oxysterols at an early stage in the sample handling procedure. The inexperienced analyst needs to keep these two points in mind when analysing oxysterols to avoid miss-identification and inaccurate quantification.

## Neurodegenerative disease including amyotrophic lateral sclerosis, hereditary spastic paraplegia type 5, Huntington's disease, Parkinson's disease and Alzheimer's disease

*Amyotrophic lateral sclerosis (ALS):* recent studies of serum/plasma and CSF of patients suffering from ALS have revealed elevated levels of free (non-esterified) cholesterol in CSF from an ALS patient group in comparison with a control group without neurodegenerative disease [32,33]. Analysis of serum revealed that both 26-HC and 3β-hydroxycholest-5-en-(25R)26-oic acid (3β-HCA), two metabolites derived from the oxidation of cholesterol by the enzyme cytochrome P450 27A1 (CYP27A1, cytochrome P450 family 27 subfamily A member 1), were reduced in the ALS patient group, suggesting dysregulation, or a reduced activity, of this enzyme is associated with ALS [32]. Importantly, Diekstra et al. [34] have identified *CYP27A1* to be a susceptibility gene for sporadic ALS in genome-wide association studies.

*Hereditary spastic paraplegia type 5 (SPG5)*, like ALS, is a form of motor neuron disease, but unlike ALS, it is a monogenic disease resulting from a deficiency in CYP7B1 (cytochrome P450 family 7 subfamily B member 1), the oxysterol 7α-hydroxylase (Figure 1) [18,35]. In contrast with ALS, both free and total (esterified plus non-esterified) 26-HC and 3β-HCA are elevated in concentration in both plasma/serum and CSF of SPG5 patients [18,21,25,35]. Interestingly, at low-µM concentrations, the levels of total 26-HC measured in serum, this molecule is found to be toxic towards a motor neuron cell line, implicating it as a toxic, disease-causing, metabolite in SPG5 [35]. Patients suffering from cerebrotendinous xanthomatosis (CTX), where CYP27A1 is deficient, like patients with SPG5, can present with motor dysfunction, and patients with both disorders are found to be deficient in the cholestenoic acid, 3β,7α-dihydroxycholest-5-en-(25R)26-oic acid (3β,7α-diHCA) [21]. Theofilopoulos et al. [21] showed that this molecule is protective to oculomotor neurons through a mechanism involving LXRs, suggesting that its deficiency in both CTX and SPG5 may be responsible, at least in part, for the motor phenotype in these diseases. Interestingly, ACOX2 (acyl-CoA oxidase-2) deficiency, an inborn error of cholesterol metabolism, like CTX can present with ataxia [36], but unlike CTX shows elevated levels in serum/plasma of C_27_ acids with unusual 25S-stereochemistry [37], including 3β,7α-dihydroxycholest-5-en-(25S)26-oic acid, the 25S-epimer of 3β,7α-diHCA, which has recently been shown to be less neuroprotective than the 25R-epimer [30]. Both 25S- and 25R-epimers are oxidised in the brain to their 3-oxo-4-ene equivalents, and 7α-hydroxy-3-oxocholest-4-en-26-oic acid has been shown to be exported out of the brain into the circulation against a concentration gradient due to the high affinity of the acid to albumin [38,39].

*Huntington's disease (HD)* is an autosomal-dominant neurodegenerative disease caused by an elongated polyglutamine repeat in the huntingtin protein [40]. HD presents in mid-life with neuropsychiatric and cognitive defects. Once diagnosed, life expectancy is usually only a further 15–20 years. An excess of 36 CAG (DNA codon for glutamine) repeats in exon 1 of the *HTT* (huntingtin) gene leads to disease penetrance and the incidence of HD is ∼10 in 100 000 in western populations [40]. In mouse models of HD, there is good evidence for reduced levels of cholesterol precursors in the striatum, the region showing atrophy in HD [41,42] and also dysregulation of cholesterol biosynthetic genes in HD cell lines and mouse models [43,44]. In their study of the YAC128 HD mouse model (transgenic line overexpressing human *HTT* with 128 glutamine residues), Valenza et al. [41] found a reduction in lanosterol, lathosterol, desmosterol (ng/mg wet weight), cholesterol (µg/mg wet weight) and 24-HC (ng/mg wet weight), the major cerebral cholesterol metabolite, in the brain of 10-month-old animals compared with wild-type animals (Supplementary Table S2). The plasma level of 24-HC was similarly reduced in 10-month-old YAC128 mice [41]. Using a second HD mouse model, R6/2, Valenza et al. [42] found levels of lanosterol to be reduced (ng/mg wet weight) in the striatum, but surprisingly, levels of cholesterol and of 24-HC did not differ in the striatum of the R6/2 mouse compared with wild-type animals. In a further study, Valenza et al. [44] confirmed that brain cholesterol and lathosterol concentrations were reduced in the 10-month-old YAC128 mouse model and in knock-in mouse models carrying the CAG expansion in the mouse *HTT* gene. In the knock-in mouse models, levels of brain lathosterol and cholesterol were reduced at the symptomatic stage [44]. In contrast with their previous studies [42], levels of cholesterol and 24-HC were found to be reduced in R6/2 mouse brain, perhaps a consequence of an age-related difference [44]. In their most recent publication, Valenza and co-workers [45] used a heterozygous knock-in mouse model of HD carrying 175 CAG repeats. While lathosterol levels in the striatum were reduced (ng/mg wet weight) in these mice at 5 (pre-motor deficit), 25 (emerging motor deficit) and 54 (significant motor deficit) weeks, cholesterol levels were only reduced (µg/mg wet weight) at 54 weeks. 24-HC levels were reduced (ng/mg wet weight) at 25 and 54 weeks.

Each of the above studies [41,42,44,45] was performed by isotope-dilution GC–MS and the values reported are for total sterols/oxysterols, i.e., the sum of esterified and non-esterified molecules (see above). This is the common reporting format for data generated using GC–MS and in the brain, where there is very little esterified sterol/oxysterol [46], can be assumed to be equivalent to free sterol/oxysterol concentration. This assumption may or may not be valid in the diseased state. 24-HC can exist as two epimers 24S-HC and 24R-HC both of which are difficult, but not impossible, to resolve by GC–MS or by LC–MS. This is probably not important for measurements made in the brain where the 24S-HC epimer is dominating but probably is where measurements are made in mouse plasma, where both epimers are evident [47]. Interestingly, the plasma levels of 24S-HC (determined as the sum of non-esterified and esterified molecule by GC–MS) are reduced in patients with HD [48,49], perhaps as a consequence of a reduced number of metabolically active neurons; however, pre-HD subjects show similar plasma levels of 24S-HC to controls [48]. As mentioned above [41,42,44,45], there is some discrepancy concerning the brain cholesterol levels in different mouse models [50]. In humans, del Toro et al. [51] found an elevation of total cholesterol (µg/mg wet weight) in caudate, a subsection of the striatum, of patients with HD and also R6/2 mouse, using enzymatic methods. The explanation for these discrepancies is not obvious but may perhaps be methodological or a consequence of differences in disease progression.

Based on their cholesterol data, Valenza et al. [52] have suggested local brain supplementation of cholesterol as a treatment for HD. They showed that nano-particle delivery of cholesterol to the brain could reduce synaptic and cognitive dysfunction in R6/2 HD mice [52]. Alternatively, Boussicault et al. [53] have suggested that CYP46A1 (cytochrome P450 family 46 subfamily A member 1), the enzyme that hydroxylates cholesterol at C-24S in the brain [2], may be therapeutic towards HD. Enhanced concentrations of CYP46A1 in neurons should increase the rate of cholesterol metabolism and in compensation, the rate of synthesis of cholesterol intermediates, noted to be reduced in HD mouse models. Boussicault et al. [53] eloquently demonstrated that adeno-associated virus (AAV) delivery of *CYP46A1* into the striatum of the R6/2 HD mouse model decreased neuronal atrophy and improved motor defects and as expected increased levels of total 24-HC (ng/mg wet weight). Levels of total desmosterol were also elevated (ng/mg wet weight), which *in vitro* was shown to be protective towards striatal neurons expressing a polyglutamine expansion [53]. These studies suggest a potential treatment of HD with CYP46A1 protein, or perhaps its metabolites.

*Parkinson's disease (PD)*: when measured as the total metabolite (sum of non-esterified and esterified), 24-HC is found to be elevated in CSF from patients with different neurodegenerative diseases [54], possibly as a result of release from dying neuronal cells, or alternatively as a consequence of CYP46A1 metabolism of released cholesterol from these cells. This contrasts to the situation in the circulation where total 24-HC is reduced, probably reflecting a reduced number of metabolically active neurons [54]. In a recent study, Björkhem et al. [55] found a significant elevation in total 24-HC in CSF from a group of patients with PD compared with a control group. Interestingly, they found a significant correlation between 24-HC and Tau in CSF from the PD patients. A similar correlation was observed between 24-HC and P-Thr^181^ Tau. Tau is a common marker for neurodegeneration and abnormal neuronal phosphorylation leads to destabilisation and increased levels of Tau and phospho-Tau in CSF. By clever use of the CYP46A1 overexpressing mouse, which has an elevated level of 24-HC in the brain, they could show that 24-HC is not the likely driving force for increased production of Tau. By investigating Tau knock-out or overexpressing mouse models, they similarly showed that levels of Tau do not increase the production of 24-HC [55]. Interestingly, *in vitro* studies with human neuroblastoma SH-SY5Y cells have shown that 24S-HC increases the levels of tyrosine hydroxylase (TH), the rate-limiting enzyme in dopamine synthesis, while 26-HC increases levels of α-synuclein and induces apoptosis [56]. Reduction in TH levels, accumulation of α-synuclein and apoptotic cell death are major hallmarks of PD. Oxidative stress is a risk factor for PD and Lewy body dementia. Bosco et al. [57] found increased levels of the cholesterol oxidation product 3β-hydroxy-5-oxo-5,6-*seco*cholestan-6-al and its aldol in the brain of patients with Lewy body dementia (0.21 µM cf. 0.09 µM in controls). 0.21 µM translates to 88 pg/mg. They used chromatographic methods for quantification and MS for identification. As with all studies of cholesterol metabolites which can be formed via non-enzymatic reactions, there is concern whether 3β-hydroxy-5-oxo-5,6-*seco*cholestan-6-al and its aldol are formed *in vivo* or *ex vivo*.

*Alzheimer's disease (AD):* cholesterol has been linked to the aetiology of AD for decades with the ε4 allele of apolipoprotein E being the most robust genetic risk factor for AD [58]. Concentrations of total 24-HC are elevated in CSF of patients with AD but are reduced in their plasma/serum [54], whereas concentrations of total 26-HC have been found to be elevated in both CSF and the brain from AD patients [59]. While CYP46A1 is normally expressed in neurons, in AD it is also expressed in astrocytes. Interestingly, one of the few factors that influence the transcription of *CYP46A1* is oxidative stress [60], perhaps as a defence mechanism against the progression of neurodegenerative disease. Björkhem et al. [61] have suggested that the balance between 24S-HC and 26-HC in the brain may affect the production of amyloid-β peptides in the brain and that brain permeable 26-HC may provide a missing link between hypercholesterolaemia and AD. Recent data indicate that the 26-HC metabolite 7α,25-dihydroxy-3-oxocholest-4-en-26-oic acid is reduced in CSF from patients with AD [62]. This may partially explain the enhanced concentration of 26-HC in AD brain being a consequence of its reduced export through metabolism. *In vivo* and *in vitro* studies in rodents have shown that 26-HC impairs neuronal morphology and hippocampal spine density and levels of the postsynaptic protein PSD95 [63]. PSD95 is one of the most abundant proteins in the postsynaptic density and is considered to be critical for proper synaptic maturation and synaptic plasticity [63]. Cedazo-Minguez and co-workers [63] have suggested inhibiting CYP27A1, the enzyme responsible for converting cholesterol to 26-HC may be a preventative strategy to reduce the risk of dementia or to improve therapies to restore neuronal function. Significantly, many existing pharmaceuticals have been shown to have inhibitory effects on CYP27A1 [64], indicating potential new treatments for AD.

While 26-HC and CYP27A1 may be associated with the cause of dementia, CYP46A1 may have a therapeutic potential towards AD. Djelti et al. [65] have shown that in the APP mouse model of AD the abundance of amyloid-β peptides increased following inhibition of *Cyp46a1* expression via AAV delivery of short hairpin (sh) RNA directed against mouse *Cyp46a1* to the hippocampus and that following treatment neuronal death was more widespread in these mice than in normal mice. Both effects were explained by an increased cholesterol content of neurons. In addition, when normal mice were injected with AAV-sh*Cyp46a1*, after 3 weeks 96% of CA3 neurons of the hippocampus contained phosphorylated Tau. Phosphorylated Tau was not detected after injection of an AAV-scrambled vector [65]. AD is characterised by both amyloid and Tau pathology. These results stimulated Burlot et al. [66] to explore the effect of AAV delivery of *CYP46A1* to the hippocampus of a mouse model of AD-like Tau pathology, with low levels of hippocampal 24S-HC. As expected, the levels of 24S-HC were normalised. Remarkably, cognitive defects and spine defects characteristic of this mouse model were rescued. The authors suggest CYP46A1 may be a target for AD therapeutic treatment [66]. In this regard, Pikuleva and co-workers [67] have suggested that the anti-HIV medication efavirenz, which activates CYP46A1, could be an anti-AD treatment.

## Hedgehog signalling

The Hh signalling pathway is important for developmental patterning. Mis-activation of the Hh pathway can lead to cancers including medulloblastoma and basal cell carcinoma. In vertebrates, there are three key proteins involved in the Hh signalling pathway, (i) Hh ligand proteins, (ii) Patched 1 (PTCH1) and (iii) SMO. Signalling proceeds when Hh ligands relieve PTCH1 repression of SMO, allowing SMO to accumulate in cilia, antenna-like projections on the surface of most cells, and activate GLi (glioma-associated oncogene) transcription factors. How SMO accumulates in cilia and is activated is not yet fully understood [68].

Oxysterols, including 20S-HC and 26H,7O-C, and cholesterol, can bind to the extracellular cysteine-rich domain (CRD) of SMO and induce Hh signalling [15,16]. To get a better understanding of the involvement of oxysterols in the Hh signalling pathway, Raleigh et al. [17] have recently profiled the oxysterol content of cilia from sea urchin embryos. Among the oxysterols enriched in cilia, they identified by LC–MS/MS (without hydrolysis), 7-oxocholesterol (7-OC, also called 7-ketocholesterol), 7β,26-diHC, 24-oxocholesterol (24-OC, also called 24-ketocholesterol) and 24S,25-epoxycholesterol (24S,25-EC). Both 7β,26-diHC and 24S,25-EC were found to bind to the CRD of SMO and activate the Hh pathway [17]. Interestingly, these two oxysterols were found to have a synergistic effect, suggesting that they may activate the pathway via multiple mechanisms. In fact, both 24S,25-EC and 24-OC activate the Hh pathway through a second binding pocket, the cytoplasmic-binding pocket, in SMO [17].

7-OC is well known to be converted to 7β-hydroxycholesterol (7β-HC) in a reaction catalysed by HSD11B1 (hydroxysteroid 11-β dehydrogenase 1, see Figure 1) [69–72], the enzyme that catalyses the conversion of cortisone to cortisol by a reduction in the 11-oxo to a 11β-hydroxy group. A pathway of bile acid biosynthesis starting with 7-OC and 7β-HC and ending with 3β-hydroxy-7-oxochol-5-enoic acid and 3β,7β-dihydroxychol-5-enoic acid and its *N*-acetylglucosamine conjugate has been suggested, with the two pathways linked by interconversion of 7-oxo and 7β-hydroxy metabolites by HSD11B enzymes [73,74]. HSD11B2 (hydroxysteroid 11-β dehydrogenase 2) is the enzyme that catalyses the oxidation of cortisol to cortisone. Interestingly, *HSD11B2* is enriched in human Hh pathway-associated medulloblastoma and Raleigh et al. [17] postulated that the Hh pathway induces the expression of HSD11B2 which promotes SMO activity by the production of oxysterols. Importantly, depletion of HSD11B2 via shRNAs attenuated Hh signalling in a cell model, as did the unselective HSD11B enzyme inhibitor carbenoxolone [17].

As previous studies had shown 26H,7O-C, like 7β,26-diHC, to bind to SMO and promote its activity [15] and as Raleigh et al. [17] showed that cells overexpressing HSD11B2 convert added 7β-HC to 7-OC, they suggested a mechanism where 7β-HC is converted to 7-OC enzymatically by HSD11B2, then CYP27A1 oxidises 7-OC to 26H,7O-C and then reactive oxygen species (ROS) reduce 26H,7O-C to 7β,26-diHC. This mechanism is perhaps a little over speculative as there appears to be no obvious source of 7β-HC to initiate the pathway and it is not clear why ROS should reduce a 7-oxo group, by the addition of two hydrogen atoms, to a give 7β-hydroxy group. Perhaps a more likely pathway to 7β,26-diHC is through a reduction in 7-OC by HSD11B1, then oxidation of 7β-HC by CYP27A1 [73]. Similarly, 26H,7O-C may be formed by oxidation of 7-OC by CYP27A1 [73]. An explanation for the promotion of SMO activity by HSD11B2 would be that 26H,7O-C is a stronger agonist to SMO than 7β,26-diHC. Whatever the mechanism for ligand formation, the discovery of 7β,26-diHC, 24-OC and 24S,25-EC as SMO agonists in cilia provides further evidence for involvement oxysterols in the Hh signalling pathway.

## Oxysterols and cancer

In addition to their link to mis-activation of Hh signalling and cancer, oxysterols, particularly 25-HC and 26-HC, have been linked to the aetiology of breast cancer [75–77], while metabolites of 5,6-epoxycholesterol (5,6-EC) have been linked to both the suppression [78] and promotion of breast cancer [79]. A recent study by Baek et al. [80] has linked 26-HC to cancer metastasis through an action on immune cells. They suggested that metastatic effects of a high-fat diet are mediated via 26-HC and showed in an animal model that metastasis could be reduced by ablation, or inhibition, of CYP27A1. It should be noted that their mice experiments were performed with rather large doses of 26-HC (20 µg/g) which induced high levels of non-esterified 26-HC in mouse plasma (0.33 µM, 134 ng/ml, measurements made in the absence of a hydrolysis step), ∼10-fold higher than normal levels of the non-esterified 26-HC in mouse or human [26,30] and more in keeping with the concentration of the sum of non-esterified and esterified molecules [26,81,82]. Baek et al. [80] also showed that the pro-metastatic actions of polymorphonuclear-neutrophils and γδ-T cells were increased by 26-HC. Interestingly, Soroosh et al. [83] have found 7β,26-diHC and 7α,26-dihydroxycholesterol (7α,26-diHC, also known as 7α,27-dihydroxycholesterol) to be activators of the nuclear receptor RORγt, driving IL-17 production in CD4^+^ Th17 cells as well as other IL-17-producing innate cells, such as γδ-T cells, and it is not inconceivable that these two molecules derived through CYP27A1 oxidation of 7-hydroxy substrates may be the drivers of the pro-metastatic actions of γδ-T cells. Similarly, as noted by Baek et al. [80], although their studies implicate 26-HC as a mediator of the pro-metastatic actions of cholesterol, the down-stream CYP27A1 metabolite 3β-HCA (Figure 1) cannot be ruled out as the bioactive metabolite. Although serum levels have been measured for 26-HC and many other oxysterols (non-esterified plus esterified), before and during breast cancer treatment in a small study and were, in fact, found to increase in women after receiving aromatase inhibitor treatment [81], there is clearly a need for a large-scale study measuring both oxysterols and cholestenoic acids in serum/plasma of breast cancer patients, to understand better the involvement of these molecules in the mechanism underlying breast cancer metastases. Nevertheless, Baek et al. [80] showed that inhibition of CYP27A1 by the small molecule GW273297X significantly reduced breast cancer colonisation of the lungs in two animal models, indicating that targeting CYP27A1 may provide a therapeutic intervention. Finally, in support of Baek's hypothesis, it is worth noting Borgquist et al. [84] found that taking cholesterol-lowering medication during endocrine therapy was associated with an increase in recurrence-free survival time and distant recurrence-free interval in postmenopausal women with early-stage hormone receptor-positive invasive breast cancer.

## Perspectives

- Oxysterols were once thought of as uninteresting intermediates in bile acid synthesis pathways.
- It is now evident that oxysterols are important bioactive molecules in multiple pathways.
- It is recognised that oxysterols are ligands to nuclear receptors, can bind to GPCRs and are allosteric modulators of NMDA receptors.
- They are also implicated as mediators of the metastatic effects of a high-fat diet and the aetiology of neurodegeneration.
- There is a current need for accurate measurement of oxysterols and inter-laboratory comparisons to define reference values for standard reference materials.

## Abbreviations

20S-HC: 20S-hydroxycholesterol
24-OC: 24-oxocholesterol
24S,25-EC: 24S,25-epoxycholesterol
24S-HC: 24S-hydroxycholesterol
25-HC: 25-hydroxycholesterol
3β,7α-diHCA: 3β,7α-dihydroxycholest-5-en-(25R)26-oic acid
3β-HCA: 3β-hydroxycholest-5-en-(25R)26-oic acid
7-OC: 7-oxocholesterol
AAV: adeno-associated virus
AD: Alzheimer's disease
ALS: amyotrophic lateral sclerosis
CRD: cysteine-rich domain
CSF: cerebrospinal fluid
CTX: cerebrotendinous xanthomatosis
EBI2: Epstein–Barr virus-induced gene 2
GC–MS: gas chromatography–mass spectrometry
GPCR: G protein-coupled receptor
HD: Huntington's disease
Hh: Hedgehog
LC–MS/MS: liquid chromatography tandem–mass spectrometry
LXRs: liver X receptors
NMDA: *N*-methyl-d-aspartate
PD: Parkinson's disease
RA: relative abundance
ROS: reactive oxygen species
sh: short hairpin
SMO: smoothened
SPG5: spastic paraplegia type 5
TH: tyrosine hydroxylase
